# The Smegma Bacillus and Urinary Tuberculosis

**Published:** 1907-12-21

**Authors:** 


### Clinical Points.

### THE SMEGMA BACILLUS AND URINARY TUBERCULOSIS.

The Ziehl-Neelsen method of staining tubercle bacilli—warm carbol fuchsin followed by decolorisation of all the structures except the bacilli by means of strong sulphuric acid—is well known, and the occurrence of bacilli stained red by this method is the clinching point in the diagnosis of many tuberculous lesions, such as phthisis.

It must be remembered, however, that notwithstanding the drastic treatment with sulphuric acid, there are two other micro-organisms besides the tubercle bacillus which retain the red stain by the Ziehl-Neelsen method. These other two bacteria are: the bacillus of leprosy and the smegma bacillus. Fortunately, in Great Britain at any rate, the bacillus of leprosy is a negligible quantity as regards differential diagnosis. The same cannot be said, however, of the smegma bacillus. It is particularly in regard to the bacteriological examination of the centrifugalised deposits from urines that the difficulty arises. The smegma bacillus may be present in the deposit, staining red in the same way that tubercle bacilli do, and the patient may very easily be thought to have a tuberculous kidney or tuberculous bladder, unless steps are taken to avoid the fallacy.

There are two main ways of getting over the difficulty, which may be summarised as: (1) The method of collecting the urine, (2) the modification in the staining method. We will deal with each of these presently, but first we will say a word or two about the smegma bacillus, its distribution and its characters.

The word smegma is apt to call to mind one particular local secretion—the sebaceous material which is apt to collect the sulcus around the corona, between the prepuce and the glans penis. This is unfortunate in a way, because it might make one think that the smegma bacillus occurred only in the male, and that if the Ziehl-Neelsen staining method showed red bacilli in the urinary deposit of a woman, these bacilli could not be smegma bacilli, and therefore must be tubercle bacilli.

The writer was perhaps not so sharp as others in his student days, and perhaps others have not experienced the same difficulty; but it was a long while before he became aware that smegma bacilli can occur in the urine of a woman just as much as they can in the urine of a man. Smegma bacilli are not even restricted in their habitat to the region of the genito-urinary organs.

They are essentially surface bacteria—that is to say, they are not found at any distance below the surface, either in tissues such as the skin, or in canals such as the urethra. They are found in chief abundance in the fatty secretions. Hence they may abound beneath the prepuce and on the glans penis. They also abound in the folds of the female genitals, particularly the labia majora et minora. In the male they are also to be found with regularity in the urethral meatus, and upon the surfaces of the thighs with which the penis comes in contact. They have also been found, however, in the ear, particularly when there has been long-standing accumulation of wax; in the sebaceous secretion all over the body; on the tongue and teeth; and in all the natural openings. Whenever there is an excessive secretion from a skin surface, the smegma bacilli may multiply locally, and an erroneous diagnosis of tuberculide of the skin might result. They abound in the secretion of ulcerated chancres, upon syphilitic condylomata, and upon similar but non-syphilitic surfaces.

Although so widespread on the surface of the body, smegma bacilli have been shown to be constantly absent from the bladder and posterior urethra. It seems clear, therefore, that a given specimen of urine can only contain those smegma bacilli which have been present in the external meatus urinarius, or upon the glans and prepuce in the male, or upon the labia in the female. It might be thought *a priori* that the number that could be washed off by the urine flowing past these surfaces at a single micturition would be so small as to be negligible. This, however, is not the case, as the experience of every clinical laboratory shows. The detection of tubercle bacilli requires such thorough centrifugalisation of the urine that even a few micro-organisms which might one would think, easily have got lost in such a bulk of urine, are collected in the deposit and transferred to the film to be stained. It is most important, therefore, in any case of doubt, or indeed in any case in which tubercle bacilli are to be looked for, to take steps to keep the smegma bacilli as far as possible out of the specimen to be examined.

Knowing as we do that the smegma bacilli collect most under the prepuce, or upon the labia, it is clear that these must be cleansed by sponging as far as possible, and then held as much as may be out of the way of the stream of urine when the latter is passed. The meatus urinarius should be cleaned in a similar way. Indeed, though it is not essential to pass a catheter, the same precautions should be taken as would be if a catheter were to be passed. Two receptacles for the urine should be at hand. The first small quantity of urine that is passed should be received into the first, and discarded—it has served to wash out the majority of the smegma baccili that have been just inside the external urinary meatus. The rest of the urine is collected in the second receptacle and kept for examination. It is as free from smegma baccilli as may be. Even if a catheter specimen is obtained, the first urine that comes through the catheter should be discarded in the same way and for the same reason.

It might be thought that, even if the staining reactions were similar, the microscopical characters of the smegma bacillus would serve to distinguish it from the turbercle bacillus. It is, in fact, possible for one who sees numbers of each kind daily to discriminate between them with fair certainty. Few of us, however, have this daily experience to help us, and we cannot rely much upon such small points as the following: That the smegma bacillus has a slightly more regular outline than has the tubercle bacillus, has more angular extremities, occurs singly or in groups of very few, and hardly ever in clumps of many, is more frail, and does not tend to appear beaded. Given a bright red bacillus of approximately the same size as the tubercle bacillus, and it must always be very difficult to be sure of distinguishing which it is from its morphological characters. It remains, therefore, to devise some method of staining the film of urinary deposit in such a way that the tubercle bacilli remain red whilst the smegma bacilli becomes decolorised, or, at least, no longer bright red. More than a score of such methods have been devised and used, but not one of them is perfect. One of the best is the following:

The films, after they have been overstained with carbil fuchsin, then decolorised with strong sulphuric acid, and after the latter has been removed, are immersed for five minutes in a solution containing 95 per cent. of alcohol and 5 per cent. of strong hydrochloric acid. The acid alcohol should then be removed with successive changes of ordinary spirit, after which the usual counterstaining with carbol methylene blue may be proceeded with. The tubercle bacilli in the finished film will still be bright red as before, while the smegma bacilli will be either partly or wholly decolorised. If completely decolorised, they will naturally not be seen at all. If only partly decolorised, they will be of a faint and dull red colour; so that the chances are that any bright red bacillus in the blue field will be a tubercle bacillus.

The smegma bacillus is a very real source of error in cases where tubercle bacilli are being sought in the urine; this applies equally whether the patient is a man or a woman. Morphologically the smegma bacillus and the tubercle bacillus are only distinguishable by those who see both frequently. The error may be reduced to a minimum if precautions are taken in regard to how the urine is collected; a catheter is not essential, but the local parts should be carefully washed before the urine to be examined is passed, and the first portion of the urine evacuated should be discarded. The error may be still further reduced if, in carrying out the Ziehl-Neelsen staining method in the laboratory, an additional step be introduced—that of immersing the film in 5 per cent. hydrochloric acid in absolute alcohol.

